# Diversity in mixed species groups improves success in a novel feeder test in a wild songbird community

**DOI:** 10.1038/srep43014

**Published:** 2017-02-23

**Authors:** Todd M. Freeberg, Shannon K. Eppert, Kathryn E. Sieving, Jeffrey R. Lucas

**Affiliations:** 1 Department of Psychology, University of Tennessee, Knoxville TN, USA; 2 Department of Ecology & Evolutionary Biology, University of Tennessee, Knoxville TN, USA; 3 Department of Wildlife Ecology & Conservation, University of Florida, Gainesville FL, USA; 4 Department of Biological Sciences, Purdue University, West Lafayette IN, USA

## Abstract

Mixed-species groups are common and are thought to provide benefits to group members via enhanced food finding and antipredator abilities. These benefits could accrue due to larger group sizes in general but also to the diverse species composition in the groups. We tested these possibilities using a novel feeder test in a wild songbird community containing three species that varied in their dominant-subordinate status and in their nuclear-satellite roles: Carolina chickadees (*Poecile carolinensis*), tufted titmice (*Baeolophus bicolor*), and white-breasted nuthatches (*Sitta carolinensis*). We found that chickadees and titmice were more likely to obtain seed from the novel feeder with greater diversity of species composition in their mixed-species flocks. For successful chickadee flocks, furthermore, the latency to obtain seed from the novel feeder was shorter the more diverse their flocks were. These results in a natural setting indicate that diversity, per se, can benefit individuals in mixed-species groups in biologically meaningful contexts such as finding food in novel places.

One of the key benefits of group living is enhanced ability to find and exploit food resources^1^. In many social species, larger groups comprise members with a diversity of personality/temperament types, and variation of types within groups may affect social organization and may improve ability to find and exploit food resources^2^. For example, a mix of reactive and proactive individuals in great tit (*Parus major*) flocks results in substantial movement of individuals while maintaining flock cohesion, facilitating effective exploration of foraging spaces^3^. Furthermore, social network analysis of mixed species flocks of great tits, marsh tits (*Poecile palustris*), and blue tits (*Cyanistes caeruleus*) revealed that individuals benefit not just from the exploration and behavior of conspecifics, but also of heterospecifics^4^,^5^. Diversity of types within social groups may provide group members with the ability to explore and exploit food resources in new and potentially risky environmental contexts.

One way in which diversity within groups is thought to benefit group members is because it enhances problem-solving ability^6^. When groups comprise members who vary in phenotype or in social network connections with one another, the group as a whole has a more diverse set of skills. The broader set of skills in diverse groups allows for more effective exploration of the problem space and greater ability to gain a solution – in comparison to more homogeneous groups, diverse groups can possess swarm intelligence^7^,^8^ and tend to be more robust (i.e., they maintain stability and functionality despite environmental changes) over time^9^. In *Apis* honeybees, for example, diverse hives containing workers from multiple sires were able to maintain consistent internal hive temperatures despite fluctuations in external environmental conditions, in comparison to hives containing workers from a single sire^10^. In experiments with human groups facing marketplace trading decisions, ethnically diverse groups were more accurate in their pricing estimates than ethnically homogeneous groups^11^. Whereas benefits of diversity have been documented in conspecific groups, it is not clear whether such benefits might extend to mixed-species groups, in which interests of members of different species are not aligned in many contexts^12^.

Mixed-species groups may form as temporary assemblages of species or as more stable associations of different species in space and time^13^,^14^,^15^. Stable mixed-species groups may establish dominance hierarchies, typically based on body-size differences among the species^13^. In these cases, we would predict that the behavior of individuals of a more subordinate species should be sensitive to the presence and number of individuals of the more dominant species. For example, the tendency of cyprinid fish to occupy a specific height in a water column varied depending upon the presence or absence of other species in each of three sympatric cyprinid species^16^. Willow tits (*Poecile montanus*) shifted their primary foraging areas to less-preferred parts of the canopy when they were in the presence of crested tits (*Lophophanes cristatus*) in mixed-species flocks, compared to when they foraged alone^17^. Mixed-species groups of animals could therefore represent an instance of greater diversity of species having a detrimental effect on behavior, especially for subordinate species in those groups.

Mixed-species groups are also often larger than conspecific groups. Increased group size – whether single-species groups or mixed-species groups – brings benefits to group members^18^. More individuals can provide greater ability to detect and exploit food (though they can also lead to greater competition over that food), and to detect and evade predators^19^. For example, downy woodpeckers (*Picoides pubescens*) spent less time being vigilant and more time foraging when in the presence of larger mixed-species groups, compared to when they were alone or with a conspecific flockmate^20^. Larger flocks of blue tits (*Cyanistes caeruleus*) and great tits (*Parus major*) were better able to solve a novel lever-pulling apparatus to obtain food, resulting in more food per individual, compared to smaller flocks^21^. Experimental manipulation of group size in captive flocks of house sparrows (*Passer domesticus*) likewise indicated that larger groups were better able to solve food-related problems, in terms of both latency to solve the problem and in the ability to exploit food sources, compared to smaller groups^22^. It is therefore possible that benefits of mixed-species groups accrue not because of increased diversity, but because of increased group size^23^. Benefits of increased group size could result from larger conspecific group sizes, but also from greater numbers of individuals of other species resulting in larger total group size.

We tested two major predictions about flexible behavior in a feeding context in wild songbird groups. First, we assessed whether the number of individuals in a group was positively associated with ability to obtain food from a novel feeder. Second, we asked whether the diversity of mixed-species groups affects ability to obtain food from the novel feeder. Diversity might be expected to increase this ability, as expected of groups with a greater range of behavioral types. Conversely, diversity might be expected to decrease this ability in specific members of the group, as expected of groups with diminished payoffs to subordinate members. We used a novel feeder test introduced to feeding stations the birds had been used to exploiting at our study sites. The novel feeder test was not particularly challenging cognitively, in terms of individuals needing to manipulate technically difficult media or apparatus. Rather, the novel feeder test related more to motivational factors of approaching and exploring, or of avoiding, a novel stimulus^24^,^25^. To be successful, birds needed to contact and explore the novel feeder to obtain the food resource, in the social context of the flock.

Our study system consisted of groups containing one or more members of three species that occur regularly in mixed-species flocks^26^: Carolina chickadees (*Poecile carolinensis*), tufted titmice (*Baeolophus bicolor*), and white-breasted nuthatches (*Sitta carolinensis*). These species provided an ideal system for testing these hypotheses as they regularly occur in the southeastern United States (where this study was conducted) and, although they are often found together in the winter months, they occasionally occur in groups composed entirely of conspecifics. Furthermore, the foraging and anti-predatory behavior of these species is sensitive to the presence and signals of the others^27^,^28^. Chickadees typically weigh 9–11 g, roughly half the mass of both titmice and nuthatches, and are the most subordinate of the three species^29^,^30^. We tested whether the ability of chickadees and titmice to obtain seed from a novel feeder was predicted best by factors assessing group size or by factors related to flock diversity, or both.

## Methods

Our study was conducted from 17 January 2016 to 19 April 2016 in eastern TN, between 0730 and 1500 (EST). We used the novel feeder test with 46 different flocks, at four locations (University of Tennessee Forest Resources Research & Education Center, 35°59′37″N, 84°13′15″W, N = 27 flocks; Norris Dam State Park, 36°13′57″N, 84°06′31″W, N = 11 flocks; Ijams Nature Center, 35°57′20″N, 83°52′06″W, N = 6 flocks, and a private residence, 36°02′46″N, 83°55′17″W, N = 2 flocks). Within each location, each study site was separated from the nearest study site by at least 400 m to ensure that different flocks were being assessed, given that most of the birds in this study were not color-banded. An earlier study with well-banded populations at these same study locations found that study sites separated by this distance represented different flocks (with 96% of marked chickadees and 90% of marked titmice being observed only at one site)^31^. We repeated the test at three study sites that had been tested earlier, but where the first flock was a conspecific-only flock and failed to obtain seed from the novel feeder. The second test at these three study sites involved species that were not tested the first time, so the novel feeder test remained novel for these other species.

The study was conducted at feeding stations that have been used for over 10 years and that represent an unpredictable food source to the birds in this study^31^,^32^,^33^,^34^. The feeding stations are simple platform feeders – each is composed of a wooden board (25 × 40 × 2 cm) attached to the top of a steel pole (1.8 m tall) set in the ground such that the wooden board is roughly 1.5 m off the ground. Each feeding station stands 1–2 m from a small tree or bush that provides perching and cover for birds using the feeder. We stocked each feeding station with ~100 g of a mix of black oil sunflower seed and safflower seed every 10–14 days in the weeks prior to, and during, our data collection period. Chickadees, titmice, and nuthatches (as well as other species) regularly use these feeding stations as food sources after they discover the presence of seed.

**Figure 1:**
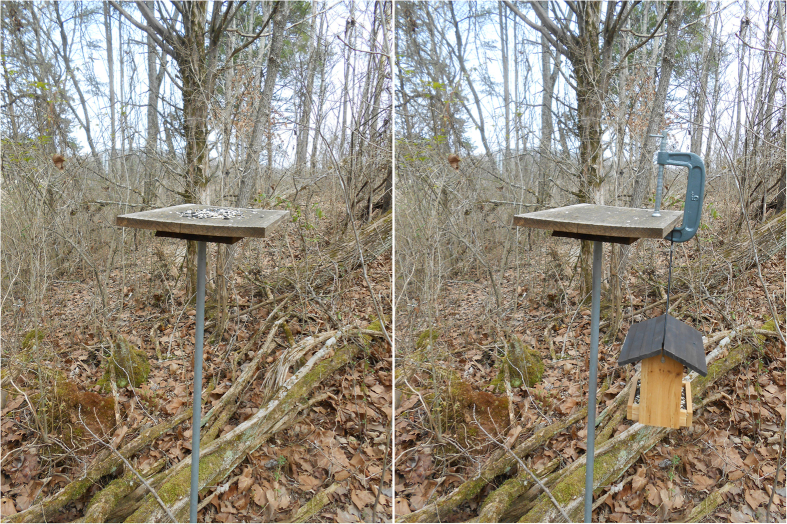
Photographs of the regular feeding station stocked with seed (left) and the novel hopper feeder attached to the empty feeding station (right). Note in the right photograph the seed visible in the hopper tray of the novel feeder. Photos by TMF.

On the morning of data collection, we stocked each feeding station with ~50 g of the sunflower and safflower seed mix. An hour or two later if we observed chickadees, titmice, or nuthatches, or some combination of the three species, we introduced the novel feeder. We first removed any remaining seed on the feeding station. We then attached a cedar hopper bird feeder (15 cm × 17 cm × 19 cm; Garden Treasures, Nature’s Way Bird Products LLC, Chagrin Falls, OH) that was ~20% filled with sunflower and safflower seed to the feeding station using a large metal C-clamp (Fig. 1). We were able to attach the novel feeder quickly (<30 sec), and such manipulation of stimuli on or near the feeders in our studies has negligible impact on the flock composition in our studies. The novel feeder hung 0.3 m below the wooden board of the feeding station. Importantly, the ample available seed in the hopper feeder was clearly visible to any bird that perched on or near the feeding stations – seed was visible either in the hopper itself or through the plastic windows that contained the seed. Considering that these study sites occurred well away from human habitation where such hopper feeders might have been present, the hopper feeder represented a novel foraging context for these flocks.

Before introducing the novel feeder test, we spent 10–15 min observing the birds exploit the seed on the regular platform feeder. This gave us time to determine the maximum number of each species we could detect at one time within a roughly 20 m radius of the feeder. We used this “maximum number observed” metric as our estimate of the real number of each species present at the feeder since the birds were not individually color-marked and since this metric is highly positively correlated with the real number of birds present at a feeder^31^. Each novel feeder test lasted 30 min. For chickadees, titmice, and nuthatches separately, we coded the latency for an individual to take the first seed from the novel feeder and the total number of seeds taken during the 30-min trial divided by the number of individuals observed (seed-taking rates). At four study sites we had two observers independently collecting data on seed-taking latencies and rates, and at 11 study sites one observer collected the same data but also video-recorded the novel feeder trial so that data on seed-taking latencies and rates could be coded later to determine inter-observer reliability. Inter-observer reliability on these measures was high (lowest Spearman correlation for titmouse seed-taking latency, N = 15, τ = 0.973).

We measured five variables that related to group size and diversity for chickadees and titmice. Conspecific flock size was the number of chickadees and number of titmice in the flocks for chickadees and titmice, respectively. Number of heterospecifics was, for chickadees, the number of titmice and nuthatches in the flock and, for titmice, the number of chickadees and nuthatches in the flock. Total flock size was the total number of chickadees, titmice, and nuthatches in the flock. Proportion of conspecifics was the proportion of the total flock composed of chickadees (for chickadees) and titmice (for titmice). The Diversity Index of the flock was assessed using the inverse Simpson index^9^,^35^ and was calculated as [(P_chickadees_)^2^ + (P_titmice_)^2^ + (P_nuthatches_)^2^]^−1^, where P is the proportion of each flock composed of chickadees, titmice, and nuthatches, respectively. The lowest Diversity Index occurred when only one species was present in the flock (diversity index = 1) and the highest Diversity Index occurred when all three species were present and had the same number of individuals (e.g., N = 2 of each, which occurred in the data set 5 times; diversity index = 3).

These five variables were correlated with one another, and so we used Principal Components Analysis with Varimax rotation to reduce variable dimension. PCAs were carried out separately for chickadees and titmice. For both species, similar principal components emerged. For chickadees, PC1 explained 60.0% of the variation and represented ‘Flock Diversity’ with loadings for proportion of chickadees (−0.949), number of heterospecifics (+0.913), and Diversity Index (+0.906). PC2 explained 32.8% of the variance and represented ‘Flock Size’ with loadings for number of chickadees (+0.958) and total flock size (+0.766). For chickadees, higher PC1 scores related to greater diversity of flocks stemming largely from more titmice and nuthatches in the flocks, and higher PC2 scores related to more chickadees and larger flocks. For titmice, PC1 explained 57.6% of the variation and represented ‘Flock Diversity’ with loadings for proportion of titmice (−0.952), number of heterospecifics (+0.906), and Diversity Index (+0.854). PC2 explained 30.0% of the variance and represented ‘Flock Size’ with loadings for number of titmice (+0.964) and total flock size (+0.769). For titmice, higher PC1 scores related to greater diversity of flocks stemming largely from more chickadees and nuthatches in the flocks, and higher PC2 scores related to more titmice and larger flocks.

‘Flock Diversity’ and ‘Flock Size’ principal component scores were analyzed for chickadees and titmice in two steps (as nuthatches rarely took seed from the novel feeder, they were not included in the analyses that follow). First, we used binary logistic regression to determine whether obtaining seed from the novel feeder (yes/no) was predicted by the principal component scores. For the subset of data involving success in the novel feeder test, we used multiple linear regression to determine whether ‘Flock Diversity’, ‘Flock Size’, or a combination of these factors, predicted latency to take the first seed from the novel feeder and seed-taking rates. We used both backward and forward stepwise regression, and both methods provided similar outcomes. Normality of residuals of regression models was verified using Kolmogorov-Smirnov tests. All statistical analyses were run in IBM SPSS Statistics Version 23. All relevant data will be made available by the corresponding author.

All our methods were carried out in accordance with published guidelines of the Animal Behavior Society, Association for the Study of Animal Behaviour, and the Ornithological Council. The experiment conducted here was approved by the University of Tennessee’s Institutional Animal Care and Use Committee (Freeberg protocol 1248).

## Results

### Study flocks

The average (±SD) number of chickadees in our study flocks was 2.2 ± 1.5 (median 2.0, mode 2.0, range 0–6). Flocks contained an average of 2.0 ± 1.5 titmice (median 2.0, mode 2.0, range 0–8). Finally, the average number of nuthatches was 0.9 ± 1.0 (median 0.5, mode 0.0, range 0–3). Average mixed-species flock size was 5.1 ± 2.5 birds (median 5.0, mode 2, range 2–11). Our study flocks comprised 34 (74.9%) groups containing more than one species, with the remaining 12 (25.1%) groups containing only a single species (7 chickadee only, 4 titmouse only, and 1 nuthatch only). Two flocks had the largest flock size we observed of 11 birds (one with 4 chickadees, 4 titmice, and 3 nuthatches; the other with 3 chickadees, 8 titmice, and 0 nuthatches). Ten flocks had the smallest flock size of 2 birds and all comprised only a single species (5 chickadee only; 4 titmouse only; and 1 nuthatch only). Nuthatches took seed from the novel feeder at only two of the 22 study sites where we observed them.

We did not obtain any indication that flocks were more likely (or quicker) to exploit seed from the novel feeder as the study progressed. For chickadees, latency to take seed from the novel feeder was not significantly associated with Julian date (for all flocks: r = +0.133, N = 40, p = 0.414; for only successful flocks: r = +0.128, N = 21, p = 0.579). For titmice, latency to take seed from the novel feeder was likewise not significantly associated with Julian date (for all flocks: r = –0.174, N = 37, p = 0.302; for only successful flocks: r = –0.305, N = 17, p = 0.233).

### Carolina chickadees

**Figure 2:**
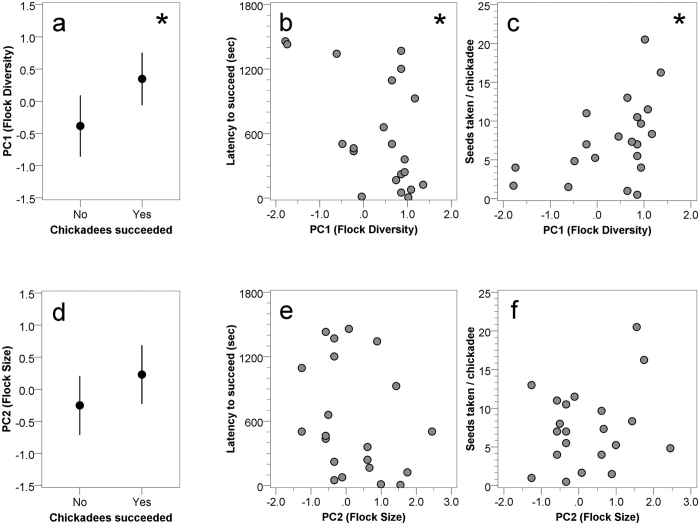
Carolina chickadee success in the novel feeder test as a function of Flock Diversity (**a**–**c**) and as a function of Flock Size (**d**–**f**). Panels a and d represent means and 95% confidence intervals for flocks that failed (No) or succeeded (Yes) at taking seed from the novel feeder. Panels b and e illustrate each successful flock’s latency to take seed from the novel feeder. Panels c and f illustrate each successful flock’s seed-taking rates (number of seeds taken in 30-min novel feeder test period divided by the number of chickadees observed at each site). Statistically significant relationships between success in the novel feeder test and flock characteristics are indicated by*.

The novel feeder test was presented to 40 flocks containing at least one Carolina chickadee. Chickadees took seed from the novel feeders at 21 of those sites. Binary logistic regression revealed that only ‘Flock Diversity’ predicted success at solving the novel feeder test (B = 0.915, SE = 0.409, Wald = 5.015, p = 0.025; Fig. 2a). For successful chickadee flocks, ‘Flock Diversity’ furthermore predicted both latency to take a seed from the novel feeder (Table 1) and seed-taking rates (Table 2). Latencies for taking seeds were shorter (Fig. 2b) and seed-taking rates were higher (Fig. 2c) for chickadees in more diverse mixed-species flocks. These two measures were correlated with one another for chickadees (r = –0.680, N = 21, p = 0.001). There was no effect of ‘Flock Size’ on success at solving the task (B = 0.617, SE = 0.382, Wald = 2.615, p = 0.106; Fig. 2d). For successful flocks, ‘Flock size’ was not a significant predictor of the latency to take a seed (Table 1 and Fig. 2e), nor did it predict seed-taking rate (Table 2 and Fig. 2f).

### Tufted titmice

**Figure 3:**
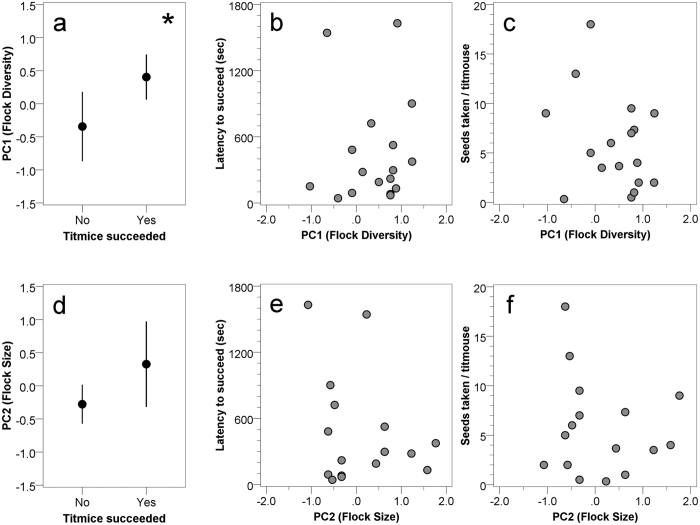
Tufted titmouse success in the novel feeder test as a function of Flock Diversity (**a**–**c**) and as a function of Flock Size (**d**–**f**). Data plotted as in Fig. 2, except from titmouse, rather than chickadee, perspective.

The novel feeder task was presented to 37 flocks containing at least one tufted titmouse. Titmice solved the novel feeder task at 17 of those sites. Binary logistic regression revealed that only ‘Flock Diversity’ predicted success at solving the task (B = 1.074, SE = 0.503, Wald = 4.559, p = 0.033; Fig. 3a). For successful titmouse flocks, however, ‘Flock Diversity’ did not predict either latency to take a seed from the novel feeder (Table 3) or seed-taking rates (Table 4; Fig. 3b and c). These two measures were not significantly correlated with one another for titmice (r = –0.387, N = 17, p = 0.125). There was a non-significant tendency for ‘Flock Size’ to be associated with success at solving the task (B = 0.799, SE = 0.442, Wald = 3.273, p = 0.070; Fig. 2d), with successful titmouse flocks tending to be larger in size. ‘Flock size’ did not predict latency to take a seed (Table 3 and Fig. 2e) or seed-taking rates (Table 4 and Fig. 2f) for successful titmouse flocks.

## Discussion

The probability that chickadees and titmice exploited seed from a novel feeder was increased by the presence of heterospecifics in their flocks. Chickadees were more likely to take seed from the novel feeder when their flocks contained proportionally more titmice and nuthatches, and when the Diversity Index of their flocks was higher. Chickadees in successful flocks also obtained seed from the novel feeder quicker and took seed at a higher rate when their flocks were more diverse. Total group size (either conspecific or total mixed-species flock size) did not predict chickadees’ abilities to solve the novel feeder test. Like chickadees, titmice were more likely to take seed from the novel feeder when their flocks were more diverse. However, flock diversity did not predict the latency to take the first seed or seed-taking rates in successful titmouse flocks. Also like chickadees, total group size did not predict titmouse ability to obtain seed from the novel feeder. For both chickadees and titmice, there were trends for larger groups to be associated with greater ability to take seed from the novel feeder in comparison to smaller groups. Perhaps a larger sample size with its greater statistical power would reveal significant effects of group size on success at the novel feeder test. Regardless, our sample size was large enough to detect significant effects of flock diversity on success at the novel feeder test.

In general, mixed-species flocks are thought to benefit flock members in terms of anti-predator abilities and vigilance^13^,^30^,^36^. Perhaps in our study, greater diversity in anti-predator behavior in diverse mixed-species flocks freed up chickadees and titmice to devote more attention to exploring the novel feeder^37^. As the socially subordinate species, chickadees are thought to be more plastic in their foraging behavior than dominant species^38^,^39^, and so would be expected to be more likely to discover a novel food source under conditions of competition in mixed-species flocks. Future studies on such food-related contexts will need to focus more on vigilance rates, social status, and competition in birds of these mixed-species flocks.

An alternative interpretation of our findings about the importance of diversity in mixed-species flocks relates to the nuclear/leader status of chickadees and titmice in these flocks and its relationships to competition and exploratory behavior. Successfully obtaining seed from the novel feeder in this study involved risk-taking on the part of the successful individuals. As mentioned above, chickadees are thought to be more opportunistic, and potentially less risk-averse, due to their subordinate status in these mixed-species flocks^38^. We might therefore have predicted chickadees to be more likely to use the novel feeder successfully than the other two species. This was certainly the case when the comparison is made to nuthatches, but we found that titmice were similarly successful at using the novel feeder in our study (52.5% of the chickadee flocks and 45.9% of the titmouse flocks were successful). The nearly complete lack of success by nuthatches in our study seems unexpected given nuthatches’ sensitivity to the behavior of chickadees and titmice^28^,^40^,^41^,^42^. However, white-breasted nuthatches are not highly competitive with chickadees or titmice for resources in mixed-species flocks. They also exhibit decreased body condition under contexts of experimental removal of chickadees and titmice in isolated woodlots, suggesting that these two nuclear species facilitate more effective foraging in nuthatches^42^. Nuthatches may not be particularly exploratory in novel contests, and relative neophobia combined with a dependence on these nuclear species for effective foraging in these flocks might therefore explain the lack of success in the novel feeder test by nuthatches. Indeed, at the two study sites where nuthatches took seed from the novel feeder, both chickadees and titmice had taken seed from the novel feeder earlier in the trial.

Future studies will also need to assess variation in movement rates of individuals in relation to flock composition. Our impression was that more diverse flocks simply stayed in the area of the novel feeder longer (though we did not measure this), and it seems likely that a greater time in relatively close proximity to the novel feeder increased the likelihood of at least one bird taking seed from the novel feeder. Perhaps a greater diversity of individuals in the area lessened neophobic responses and arousal levels in some of the birds, enough for them to use the novel feeder successfully. The lack of association between Julian date and success at taking seed from the novel feeder adds further support to argument that our study flocks were independent of one another. Had there been significant movement of individuals from one site to nearby sites, we might have expected to see decreased latencies to take seed from the novel feeder with increased Julian date if later flocks were composed partly of individuals from earlier, successful flocks.

The diversity of mixed-species flocks benefited chickadees and titmice in solving a novel feeder test. Mixed-species flocks are proposed to effect greater foraging success in individuals seeking food through processes such as social learning and flushing prey items from cover^43^,^44^. Although we did not assess social learning or social facilitation of feeding from the novel feeder in our study, we would expect these processes to be occurring in flocks facing this task^3^,^20^,^45^. Similar processes are thought to play in a diversity of human groups in the context of problem solving^6^. In human groups, diversity typically improves problem-solving abilities^6^,^46^,^47^. In cases where diversity fails to enhance problem solving in human groups, it is generally thought that lack of communication among group members is a key reason for the failure. In the birds of our mixed-species flock, there are no such barriers to communication – individuals in mixed-species flocks with chickadees and titmice respond adaptively to their calls (although this has been experimentally demonstrated so far only in predator mobbing contexts)^28^,^41^,^48^. Our future work will assess the signaling that occurs in these groups in more novelty response tests, as well as in more technical problem-solving tasks. How do the signals and cues of individuals in diverse mixed-species flocks differ from signals and cues of individuals in single-species flocks? We know that diversity of signals increases with increases in group size in chickadees^49^. We also know that in other species, larger groups result in greater problem-solving ability for individuals in those groups^21^,^22^. We now have added what is, to our knowledge, the first evidence in a natural setting that diversity, per se, can benefit individuals in mixed-species groups in biologically meaningful contexts such as finding food in novel places.

## Additional Information

**How to cite this article:** Freeberg, T. M. *et al*. Diversity in mixed species groups improves success in a novel feeder test in a wild songbird community. *Sci. Rep.* **7**, 43014; doi: 10.1038/srep43014 (2017).

**Publisher's note:** Springer Nature remains neutral with regard to jurisdictional claims in published maps and institutional affiliations.

## Figures and Tables

**Table 1:** Backward linear regression models for seed-taking latencies in context of novel feeder for successful Carolina chickadees.

Model	Predictor	Unstandardized coefficients			
		B	SE	t	p
1	Intercept	732.50	105.87	6.92	0.001
	Diversity	−294.95	111.20	−2.65	0.016
	Size	−120.10	99.03	−1.21	0.241
2	Intercept	709.82	105.49	6.73	0.001
	Diversity	−308.53	112.00	−2.76	0.013

Diversity represents the Flock Diversity principal component and Size represents the Flock size principal component.

**Table 2:** Backward linear regression models for seed-taking rates in context of novel feeder for successful Carolina chickadees.

Model	Predictor	Unstandardized coefficients			
		B	SE	t	p
1	Intercept	6.42	1.10	5.82	0.001
	Diversity	2.61	1.16	2.15	0.037
	Size	0.95	1.03	0.92	0.369
2	Intercept	6.60	1.08	6.11	0.001
	Diversity	2.71	1.15	2.37	0.029

Diversity represents the Flock Diversity principal component and Size represents the Flock size principal component.

**Table 3:** Backward linear regression models for seed-taking latencies in context of novel feeder for successful tufted titmice.

Model	Predictor	Unstandardized coefficients			
		B	SE	t	p
1	Intercept	512.11	153.42	3.34	0.005
	Diversity	−43.30	196.06	−0.22	0.828
	Size	−121.03	103.47	−1.17	0.262
2	Intercept	492.50	121.05	4.07	0.001
	Size	−114.59	96.07	−1.19	0.251
3	Intercept	455.00	118.44	3.84	0.001

Diversity represents the Flock Diversity principal component and Size represents the Flock size principal component.

**Table 4:** Backward linear regression models for seed-taking rates in context of novel feeder for successful tufted titmice.

Model	Predictor	Unstandardized coefficients			
		B	SE	t	p
1	Intercept	6.89	1.50	4.61	0.001
	Diversity	−2.21	1.91	−1.16	0.267
	Size	−0.21	1.01	−0.21	0.839
2	Intercept	6.78	1.35	5.04	0.001
	Diversity	−2.10	1.77	−1.18	0.255
3	Intercept	5.93	1.15	5.14	0.001

Diversity represents the Flock Diversity principal component and Size represents the Flock size principal component.
